# Acute bilateral granulomatous anterior uveitis as an extra-hepatic manifestation of hepatitis A virus (HAV) infection: a case report

**DOI:** 10.1186/s12348-020-00210-6

**Published:** 2020-08-27

**Authors:** Ali Azimi, Mohammad Shirvani, Shahla Hosseini, Vahid Bazojoo, Nasrin Masihpoor, Sahar Mohaghegh, Seyed Mohammad Sadeghi

**Affiliations:** 1 Poostchi Ophthalmology Research Center, Shiraz University of Medical Sciences, Shiraz, Iran; 2 Infectious Ophthalmologic Research Center, Ahvaz Jundishapur University of Medical Sciences, Ahvaz, Iran; 3 Optometry Department, School of Rehabilitation, Shahid Beheshti University of Medical Sciences and Health Services, Tehran, Iran

**Keywords:** Uveitis, Hepatitis A virus, Infection, Visual acuity

## Abstract

Anterior uveitis (AU) is the most common form of uveitis. The differential diagnosis of AU is broad, ranging from infectious etiologies to autoimmune causes. However, approximately half remain idiopathic. Infections are the vision-threatening causes of AU which should be ruled out by history taking and detailed physical examination combined with guided work up. We report a rare case of bilateral granulomatous AU following hepatitis A virus (HAV) infection in an immunocompetent patient. A 35-year-old male presented to our center with a chief complaint of pain and redness in both eyes 3 days prior to the presentation. The patient’s medical and drug history was unremarkable. He had a history of river water consumption 20 days prior to presentation. The patient was diagnosed with acute bilateral granulomatous AU. All routine work up to investigate the etiology of the disease was unremarkable, except for the serology of acute HAV infection, which was positive. The patient was managed with the topical steroid and cycloplegic for 2 weeks with no recurrence at one-year follow up. Extra-hepatic complications of HAV were reported in previous studies including arthritis, urticaria, myocarditis, nephritis, and myositis. The mechanism of extra-hepatic complication of hepatitis A is unknown; however, immune-complex deposition is most likely the etiological cause. Our report represents a rare case of sudden onset with limited duration granulomatous AU as a presenting manifestation of HAV infection. Previous studies do not provide a direct evidence of granulomatous AU associated with the HAV infection.

## Introduction

Uveitis is a common inflammatory ocular disease which can lead to visual impairment. Idiopathic AU is the most common form of AU across the world. Various factors such as patient’s age, race, gender and disease characteristics such as laterality and histopathology, are helpful to identify the possible etiology [1]. Infections are the vision-threatening causes of AU which should be ruled out by history taking and physical examination combined with guided work up [2]. Uveitis is a rare complication of acute viral hepatitis B and C which has not been reported as a presentation or extra-hepatic manifestation of hepatitis A virus (HAV) infection so far [3–5]. Hepatitis A is a self-limited viral infection with an average 30 days (range 15 to 49 days) of incubation period [6]. In this case study we report a rare case of bilateral granulomatous AU as an extra-hepatic manifestation of HAV infection in an immunocompetent patient.

## Case report

**Fig. 1:**
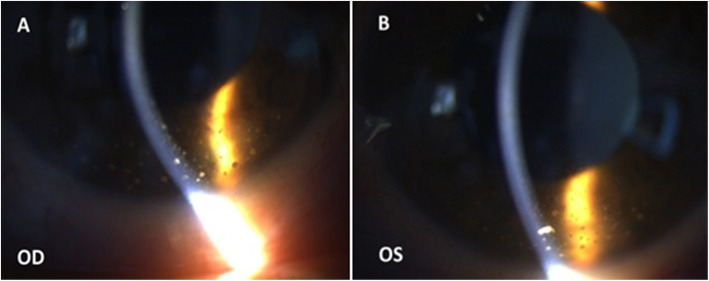
Slit lamp photos of both eyes (**a** and **b**), slit beam illumination shows multiple variable size mutton-fat keratic precipitates inferior to the cornea

**Fig. 2:**
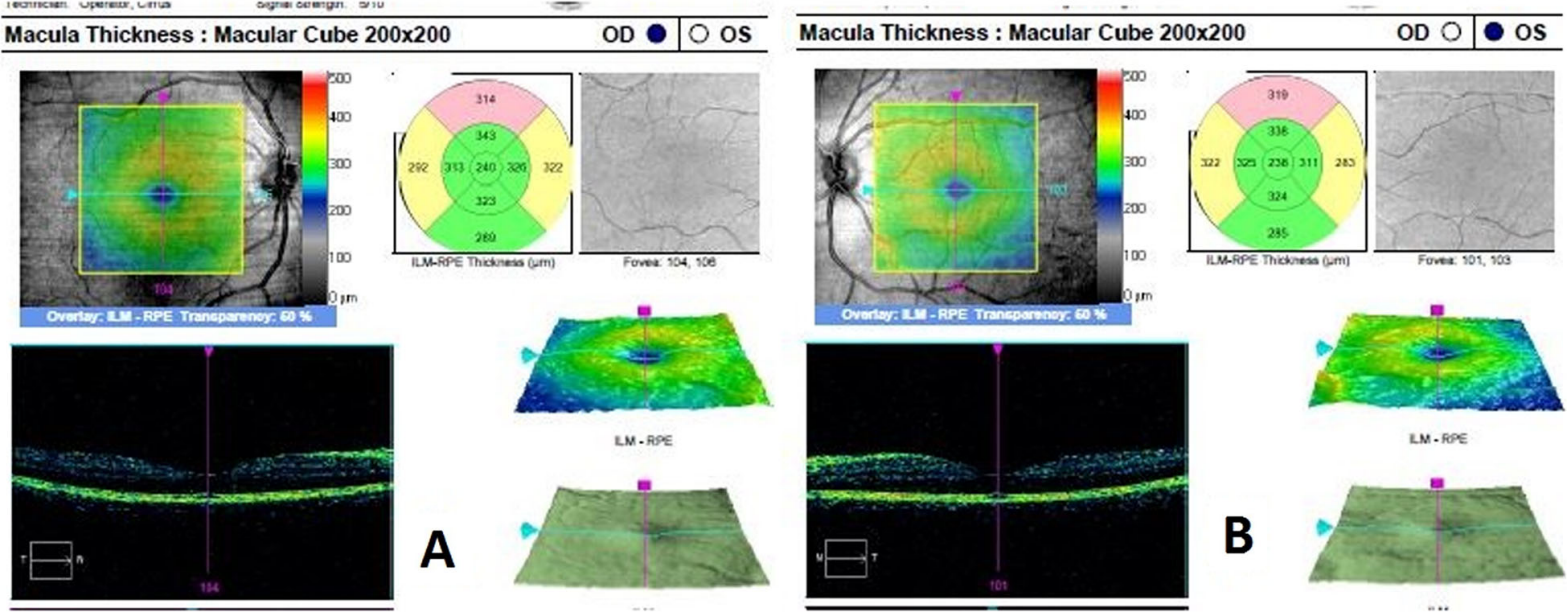
Optical coherence tomography of the macula, Infra-red fundus images and macular thickness map images of both eyes (**a** and **b**) are normal

A 35-year-old male presented to our center (Khalili Hospital, Shiraz, Iran) with a chief complaint of pain and redness in both eyes 3 days prior to the presentation. The patient’s medical history was unremarkable. He had a history of river water consumption 20 days prior to the presentation. He had no symptoms and signs of fever, diarrhea, jaundice, abdominal pain, morning stiffness, articular pain, and oral aphthous lesions. The ocular pain and redness started suddenly with mild photophobia 3 days before his presentation. The best corrected visual acuity (BCVA) was 20/25 in the right eye and 20/22 in the left eye. Intraocular pressure was 12 and 14 mmHg in the right eye and left eye, respectively. Slit lamp examinations of both eyes revealed conjunctival injection, variable-sized mutton-fat keratic precipitates in the inferior cornea (Fig. 1a,b), and + 3 anterior chamber (AC) cells. The iris, lens, and vitreous were normal. No hypopyon, posterior synechiae and iris atrophy were noted. Fundus examinations were unremarkable in both eyes. The macular optical coherence tomography in both eyes was also normal (Fig. 2a,b). Bilateral granulomatous acute AU was diagnosed.

Three days after ocular presentation, the patient developed low-grade fever, non-productive cough, nausea, vomiting, and yellowish sclera. Routine laboratory tests including complete blood count, erythrocyte sedimentation rate, fasting blood sugar, lipid profile, creatinine, thyroid function tests, and calcium and vitamin D levels were all within normal; only the liver function tests revealed increased levels of alanine aminotransferase (ALT) (183 U/L), and aspartate aminotransferase (AST) (131 U/L). Other investigations showed raised bilirubin (total bilirubin 7.3 mg/dl, and direct bilirubin 4.6 mg/dl) and alkaline phosphatase (ALP) (160 U/L) levels. The infectious investigations including Mantoux test, interferon-gamma blood test, human immunodeficiency virus antibody, syphilis serology tests including venereal disease research laboratory and fluorescent treponemal antibody absorption, hepatitis B surface antigen, hepatitis B core antibody, hepatitis C virus (HCV) antibody, anti-leptospira immunoglobulin (Ig) M antibody, serology of Lyme disease, and blood and urine cultures were all unremarkable except for a positive serology of HAV IgM antibody. The rheumatologic tests including anti-nuclear, anti-neutrophil cytoplasmic, anti-mitochondrial, anti-smooth muscle, anti-La and anti-Ro, anti-liver/kidney microsomal type 1, anti-cyclic citrullinated peptide, anti-phospholipid, anti-β2 glycoprotein, anti–thyroid peroxidase, and anti-thyroglobulin antibodies, rheumatoid factor, and serum angiotensin converting enzyme level were unremarkable. The human leukocyte antigen (HLA) B27, and HLA B51 tests were negative. Chest X-ray radiography was normal without hilar lymphadenopathy. The patient was treated with topical corticosteroid eye drop (prednisolone 1%, Sina Daru Co.) six times a day and cycloplegic eye drop (homatropine 2%, Sina Daru Co.) three times a day for 2 weeks. The HAV infection was managed with supportive care including replacement of fluid. The ocular pain decreased, AC cells disappeared, and the BCVA improved to 20/20. There was no relapse of AU at the 1 year follow-up. Liver enzymes, and bilirubin returned to the normal levels (ALT:21 U/L, AST:16 U/L, ALP:112 U/L, total bilirubin:0.8 mg/dl, and direct bilirubin:0.1 mg/dl), and HAV serology test was negative for anti-HAV IgM and positive for anti-HAV IgG within 12 months following the treatment.

Written informed consent was obtained from the patient for publication of this case report and accompanying images.

## Discussion

The incidence and prevalence of uveitis depends on age, gender, anatomic position of the inflammatory process (anterior, intermediate, posterior uveitis, pan-uveitis), histopathology (granulomatous, non-granulomatous), course of an inflammatory process (acute, chronic, recurrent), and etiology (infectious, non-infectious). The most common anatomic form of uveitis is AU [1, 2]. Although AU has a diverse spectrum of infectious and non-infectious etiology, approximately 50% remain idiopathic [1, 2, 7, 8]. The clinical and etiologic patterns of uveitis in Northeastern Iran were evaluated by Hosseini et al. They reported that pan-uveitis is the most common clinical pattern with an idiopathic etiology. Moreover, toxoplasmosis was among the most common infectious causes of uveitis [9]. Hepatitis viruses are one of the infective agents considered in the pathogenesis of uveitis. The association between viral hepatitis and uveitis has been proposed in a few studies, most of which investigated the role of hepatitis B and C in the pathogenesis of uveitis. These studies noted that deposition of circulating immune complexes, physiologic immune reaction against an infection within the eye, and complement-mediated immune activation may lead to extra-hepatic manifestations such as glomerulonephritis, uveitis, and polyarteritis nodosa [3–5]. Tien at el. in a cohort study on the relationship between uveitis and the different types of viral hepatitis concluded that patients with hepatitis B virus and HCV co-infection have the highest risk of uveitis. Also, They reported only one case of uveitis among 82 cases of isolated HAV infection (rate:19.44 per 10,000 person-years) [5]. The extra-hepatic manifestations which reported to be associated with HAV infection include glomerulonephritis, acute renal failure (ARF), myositis, acute pancreatitis, arthritis, pleural or pericardial effusion, myocarditis, autoimmune thrombocytopenic purpura, erythematous maculopapular rash, and parotitis. In these conditions, deposition of the immune-complex in various organs such as the skin, joints, kidneys, and muscles leads to immune system activation [6, 10–12]. Bhatt et el. noted that direct invasion of HAV into the kidney tissues as well as immune-related mechanisms may lead to ARF [11]. In our literature review, previous reports of ophthalmic manifestations associated with HAV infection were not found. Stangos et al. reported a case of multiple evanescent white dot syndrome (an inflammatory chorioretinal disorder) 10 days after receiving simultaneous formalin-inactivated HAV and live-attenuated yellow fever vaccine. They concluded an autoimmune etiology contributing to this ocular inflammation [13]. The antigen-antibody complex deposition and granulocyte aggregation in the highly vascularized uveal tract and choroid may lead to activation of the immune system. The expression of pro-inflammatory cytokines, (e.g. interleukin 1) and HLAs, (e.g. HLA-DR) in the intraocular tissues secondary to systemic viral infection or inflammation may also result in uveitis [14–16]. However, the primary underlying immune-related mechanisms that compromise the intraocular immune privilege and blood–ocular barrier are not fully understood [15, 16]. Some studies have shown extra-hepatic manifestations of HAV infection occurred following non-specific symptoms at presentation [6, 10, 12]. In our study, the patient presented with acute AU 3 days before presentation of typical systemic symptoms of HAV infection. Bhatt at el. reported a 34 year-old man with acute parotitis and facial skin rash as the manifestations of HAV infection 6 days prior to any hepatic presentations [11] These findings suggest that extra-hepatic manifestations of HAV infection may occur before (during incubation period) or after the presentation of systemic and hepatic signs secondary to deposition of circulating immune-complex in various organs.

To the best of our knowledge, our case is the first report of granulomatous AU associated with HAV infection. The pathophysiology of the ocular involvement of HAV infection is unknown. The probable mechanisms for ocular involvement may be immune-related such as antigen-antibody complex deposition and activation of complement pathway secondary to systemic viremia. Therefore, the administration of topical steroid and other anti-inflammatory drops can control the ocular inflammation associated with the disease. We used topical prednisolone to manage the inflammatory process as well as topical cycloplegic drop for pain relief and prevention of synechiae formation. We did not perform anterior chamber sampling although it could help in the detection of direct HAV-induced uveitis or immune-related uveitis [16]. This study suggests HAV infection should be considered in acute anterior uveitis (AU) patients who have had a history of travelling to areas of high endemicity, with limited access to safe water sources, and exposure to an infected person.

## Conclusion

Hepatitis A virus infection might lead to ocular involvement. The exact mechanism of the ocular involvement following HAV infection is unclear. The HAV infection may contribute to the etiology of AU through an immune complex mediated reaction.

## Data Availability

All data generated or analyzed during this study are included in this published article (and its supplementary information files).

## Abbreviations

HAV: Hepatitis A virus
AU: Anterior uveitis
BCVA: Best corrected visual acuity
AC: Anterior chamber
U/L: Unit per liter
ALT: Alanine aminotransferase
AST: Aspartate aminotransferase
mg/dl: Milligram per deciliter
ALP: Alkaline phosphatase
HCV: Hepatitis C virus
Ig: Immunoglobulin
HLA: Human leukocyte antigen
ARF: Acute renal failure
